# Ultra pure high molecular weight DNA from soil for Nanopore shotgun metagenomics and metabarcoding sequencing

**DOI:** 10.1016/j.mex.2024.103134

**Published:** 2024-12-28

**Authors:** Arthur Cousson, Anne-Laure Pablo, Laurent Cournac, Gabin Piton, Damien Dezette, Agnès Robin, Elisa Taschen, Laetitia Bernard

**Affiliations:** a IRD, UMR Eco&Sols, INRAE, CIRAD, Institut Agro, Université Montpellier, Montpellier, France; b INRAe, UMR Eco&Sols, IRD, CIRAD, Institut Agro, Université Montpellier, Montpellier, France; c CIRAD, UMR Eco&Sols, INRAE, IRD, Institut Agro, Université Montpellier, Montpellier, France

**Keywords:** Solvent free, Bacterial cells, Long fragments, Community DNA, Nycodenz, MP kit, Metabarcoding, Metagenomics - Oxford nanopore nanopore sequencing, Ultra Pure HMW DNA extraction and purification from soil for Nanopore sequencing

## Abstract

Soil microbes are among the most abundant and diverse organisms on Earth but remain poorly characterized. New technologies have made possible to sequence the DNA of uncultivated microorganisms in soil and other complex ecosystems. Genome assembly is crucial for understanding their functional potential. Nanopore sequencing technologies allow to sequence long DNA fragments, optimizing production of metagenome-assembled genomes compared to short-read technology. Extracting DNA with a very high purity and high molecular weight is key to get the most out of this long read technologies. Here we present two extraction protocols to get DNA with high purity. First protocol is optimized to reach DNA quality suiting Nanopore shotgun metagenomics. It uses a non-toxic centrifugation gradient to separate bacterial cells from soil to extract DNA directly on cells. The median length of the acquired DNA sequences (N50) was 3 to 7 times greater than previously published in the literature, achieving an N50 of ∼14 kb. The other, a modification of a commercially available MP Biomedical DNA extraction kit, yielded high-purity DNA for full-length 16S Oxford Nanopore metabarcoding, with an N50 of ∼8 kb. The MP-based protocol achieves higher yields of ultra-pure DNA compared to the Nycodenz protocol, at the expense of shorter fragment lengths.

## Graphical abstract

**Figure:**
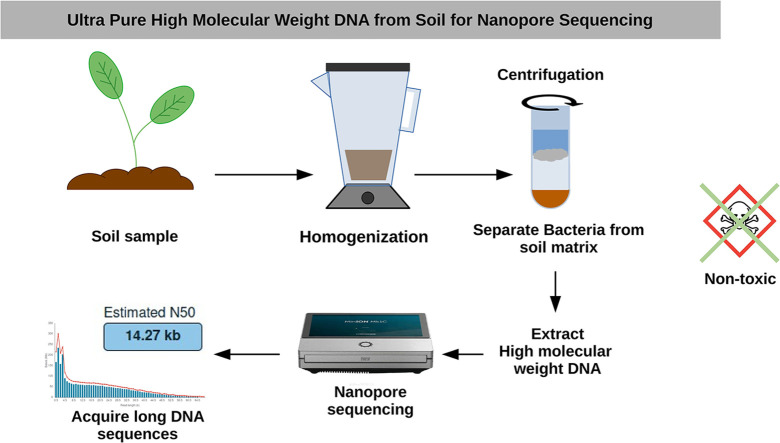

Specifications table

**Table:** 

Subject area:	Biochemistry, Genetics and Molecular Biology
More specific subject area:	*Ultra pure high molecular weight DNA extraction*
Name of your protocol:	*Ultra Pure High Molecular Weight DNA from soil for Nanopore shotgun metagenomics sequencing*
Reagents/tools:	Nycodenz gradient solution Nycodenz® is a non-ionic tri-odinated derivative of benzoic acid with aliphatic hydrophilic side chains. The systematic name of Nycodenz is 5- (N-2, 3-dihydroxypropylacetamido)−2, 4, 6-tri-iodo-N, N'-bis (2, 3 dihydroxypropyl) isophthalamide. Nycodenz solution (Axis-Shield, Oslo, Norway) at 1.3 g.mL^−1^ density was prepared by stirring 8 g to 10 mL of MilliQ H_2_O. Warming to 50 °C of the solution allows the powder to dissolve. [1] Sodium hexametaphosphate solution Solution was prepared by dissolving sodium hexametaphosphate in MilliQ ultra pure H_2_O for a final concentration of 0.2 %. Sodium hexametaphosphate solution acts as a dispersing agent breaking down clay and separating bacteria from it. Solution was filtered with 0.2 µm sieves for sterilization.
	Lysis buffer Lysis buffer was prepared according to recipe [2]: Trizma Base 100 mM pH 8; EDTA 100 mM pH 8; NaCl 100 mM; SDS 2 %; MilliQ ultra pure H_2_O. The solution was sterilized by filtration through a 0.2 µm sieve. Potassium acetate solution Solution was prepared with a final concentration of 3 M pH 5.5. Potassium acetate solution precipitates salts, proteins and other pollutants. The solution was sterilized by filtration through a 0.2 µm sieve. Washing ethanol solution A 70 % volume solution of ethanol was prepared with molecular biology grade ethanol and MilliQ ultra pure H_2_O. Sterile Sodium Chloride solution 0.8 % sodium chloride solution was prepared with NaCl and MilliQ ultra pure water. Then filtered with 0.2 µm sieve for sterilization. **RNase Cocktail Enzyme Mix (ThermoFisher, AM2286)** **AMPure XP Reagent, SPRI Reagent Product No: A63880 – Beckman Coulter** Blender Centrifuge with swing out rotor
Experimental design:	*The idea is to separate bacterial cells from the soil matrix and gently extract DNA, aiming to preserve the longest fragments possible. The product should be ultra pure to allow best sequencing results. Long DNA fragments enhance and facilitates MAGs assembly and genome annotation.*
Trial registration:	*None*
Ethics:	*None*
Value of the Protocol:	• High molecular weight DNA recovery • Ultra pure DNA quality • Non-toxic for laboratory workers and environment • Unexpensive

## Protocol descriptions

### Background

Soil microbial communities are incredibly diverse with a bacterial diversity predicted around 4 × 10^9^ taxa in soil [3]. the fungal diversity follows smaller predictions with 6.2 × 10^6^ species [3]. They are yet largely uncharacterized despite having a central role in plant growth and organic matter dynamics and therefore terrestrial biogeochemical cycles [4]. Most of the current knowledge about the specific functions of microorganisms is based on pure strains grown in the laboratory, which represent only a small fraction of the total species [5]. Cultural approaches introduce strong biases in our characterization of microbial communities as it focus on few microbes that we are able to grow in the laboratory [6]. Recent genomic methods offer the possibility to study microbial genomes directly from the soil DNA extracts, giving access to uncultured microbes [7]. Metagenomic-assembled genomes (MAGs) can be reconstructed while they also have limitations linked to bioinformatics process and reference databases [8]. Nevertheless, once a MAG or contigs are obtained for a microbe, it allows to characterize its genes, their functions if they are known and to link this functional information with its taxonomy (usually attributed based on marker genes like 16S or single copy core gene). Such taxa-function links can then be very useful to improve functional inference from taxonomic composition of the community obtained with classical metabarcoding.

Gene annotations and MAG assembly benefit from long sequences [[9], [10], [11]]. These sequences help resolve the synteny of repetitive elements [12], circularize bacterial genomes [13], recover circular plasmids [14], distinguish closely related strains within a metagenome [15], and recover full length genes open reading frames [12,16], among other advantages. However, until now, most accessible sequencing technologies (Sanger, Pyrosequencing, Illumina etc.) only allowed the analysis of small DNA fragments (ex : 2 × 250 bp Illumina), generating heavy downstream assembly processes with low contiguity and difficulties linked to repeated sequences [17]. Oxford Nanopore sequencing Technologies (ONT) now offers to generate long-reads [18], with no theoretical limit. However, this technology requires ultra pure high molecular weight (HMW) DNA [19]. Extraction of HMW DNA have already been optimized for the extraction of DNA from organisms [20], but it remains non-trivial for environmental DNA [19]. Soil humic acids and metallic ions are deleterious to Nanopore proteins and *in fine* to overall sequencing quality. Here we present two DNA extraction protocols, both optimized to reach high DNA purity requirement, with one (Nycodenz based DNA extraction) inspired from Lindahl and Bekken [1] refined for obtaining very long fragments relevant for metagenomics and the other (MP Biomedical kit based DNA extraction) optimized for full length 16S metabarcoding Nanopore sequencing. Differences and sequencing results from the two protocols are summarized in Table 1.

**Table 1:** Summary table comparing the characteristics of the two protocols. N50 (in kilobases) represents the median length of the acquired DNA sequences. The 260/280 and 260/230 ratios, based on optical absorbance, are indicators of DNA purity, with optimal values for ultrapure (UP) DNA being approximately 1.8 and 1.9–2.0, respectively. Nycodenz based protocol (1) produces ultra pure high-molecular-weight (HMW) DNA but requires 200 times more starting material compared to MP based protocol (2).

**Protocol**	**Sample mass (g)**	**[DNA] ng.µl**^−1^**(100µl elution)**	**260/280**	**260/230**	**Yield** (µg of DNA / g of soil)	**N50 (kbp)**	**Longest Sequence**	**Application**
**1 – Nycodenz based**	100	30–50	1.8	1.8–1.9	0.05 – 0.1	14.27	142 kb	Metagenomics
**2 – MP based**	0.5	100–120	1.8	1.9–2	20–30	8	80 kb	Metabarcoding Metagenomics

## Protocol 1 – Nycodenz based DNA extraction

Separation of cells from soil particles

1. Soil samples : ∼100 g of frozen soil (−20 °C) or fresh soil are needed for cells separation.
2. Homogenize soil sample in a blender with 150 mL of sodium hexametaphosphate 0.2 % for 1 min. Other surfactant (tween, pyrophosphate…) allowing to separate bacteria from mineral might also be used but were not tested.
3. Divide the suspension into four 50 ml centrifuge tubes and spin at 700 x g for 15 min (10 °C) to eliminate coarse soil particles. *Lower spinning speed might eventually lower cells loss, but was not tested.*
4. Collect supernatant and filter using a sterile gauze (100 µm) or similar. *This step leads to eliminate floating debris that would still be present in supernatant after the first centrifugation.*
5. Centrifuge at 7 500 x g for 20 min (10 °C). *This step allows clay and microbes to pellet.*
6. Resuspend the pellet in sterile 0.8 % sodium chloride solution by vortexing 15 s. *Adjust the solution volume to the pellet size.*
7. Equally divide your cells suspension in two centrifuge tubes above 10 mL of 1.3 g.mL^−1^ Nycodenz solution. Take care not mixing phases. *If the aqueous phase and the Nycodenz phase mix, the density of the Nycodenz solution will be affected and the cells may not separate properly from the clay.*
8. Centrifuge at 14 600 x g for 40 min (10 °C) using a swing-out rotor [1]. Swing out rotors allow the cells to be pelleted at the Nycodenz-water interface and not at the edge like fixed-angle rotors.
9. A white band of bacterial cells is obtained at the interface of Nycodenz and initial cell suspension. Carefully recover the cells in new tubes. *To facilitate this step, place the tube in front of direct light, allowing to easily distinguish the phases and the cells.*
10. Add 5 mL of sterile ultra pure water to the cells (washing step).
11. Centrifuge at 7 500 x g for 20 min (10 °C), discard supernatant
12. Repeat steps 10 & 11. *If some volutes appear, this means that another washing step is required to eliminate residues of Nycodenz solution.*

**DNA extraction**

13. Gently resuspend the pellet in 1 mL of lysis buffer. *Several lysis buffer recipes have been tested. (some including Proteinase* K *based lysis buffer [**21**]) with no impacts on results.*
14. Incubate 30 min at 70 °C. Gently flick the tube at 15 min and 30 min. If *possible, the thermal block is preferred to the water bath to avoid contamination.*
15. Centrifuge at 7000 x g for 5 min at room temperature.
16. Transfer supernatant in a new 1.5 mL microtube and add 100 µl (= 1/10 vol) of sodium acetate 3M
17. Incubate 10 min on ice. White cloud of precipitated salts and proteins will form.
18. Centrifuge at 7500 x g for 5 min at 4 °C. Transfer supernatant to a new 2 mL microtube.
19. Add 900 µl of −20 °C isopropanol. Invert tubes a few time to ensure good mixing.
20. Incubate at −20 °C overnight.
21. Centrifuge at 13,000 x g for 30 min at 4 °C
22. Carefully discard supernatant without disturbing the (mostly invisible) pellet. Add 400 µl of -20 °C 70 % ethanol solution. *The pellet might appear vitreous or transparent.*
23. Centrifuge at 13,000 x g for 5 min at 4 °C.
24. Repeat steps 22–23. Carefully discard supernatant.
25. Let the pellet dry before next step.
26. Resuspend the (mostly invisible) pellet in 100 µl of sterile ultra pure water. A 10 min at 60 °C will speed up the process.

**DNA purification**

27. Add 0.5 µl of 10 mg.mL^−1^ RNase Cocktail Enzyme Mix (ThermoFisher, AM2286) solution to the eluate, incubate at 37 °C for 30 min*. This step leads to the elimination of co-extracted RNA.*
28. Add 180 µl of AMPure XP beads suspension to the eluate (= 1.8 ratio) – Incubate 10 min at room temperature. *Warming the bead suspension to 30* °*C beforehand can improve its efficiency. Resuspend the beads completely before pipetting. Humic acids being in competition with DNA for binding to the magnetic beads, the eluate must be as clear as possible. Note that sparQ PureMag Beads (QuantaBio) works with similar efficiency (ratio 1.8)*
29. *Place the tube on a magnetic rack until the supernatant clears due to the migration of beads on the wall of the tube. Magnetic rack, can be purchased or homemade 3D printed after downloading the 3D model designed for this application (* *https://www.tinkercad.com/things/5kDi01FuJtp* *). This model fits with Ø10 mm and 2 mm thick powerful magnets (such as N48, N52, neodymium magnets).*
30. Leave the tube on the rack, open the cap, remove supernatant . Add 200 µl of molecular biology grade 70 % ethanol solution without disturbing the beads. Wait 30 sec before processing.
31. Repeat four times steps 29–30. Then let ethanol residue dry with open cap but not to the point of cracking.

**DNA elution**

32. Gently resuspend the beads in 50 µl of sterile ultra pure water. *Resuspension is a good indicator of success. The beads should take the form of a string of pearls. At the opposite, immediate resuspension of the beads may indicate a low amount or a highly fragmented state of DNA.*
33. Incubate tubes with open caps for 15 min at 37 °C to eliminate ethanol residues. E*thanol might impair future sequencing reactions. Thermal block lid should be closed to avoid contamination.*
34. Place the tube back on the magnetic rack until the solution clears.
35. Carefully transfer the supernatant into a new DNA low-biding microtube. HMW DNA fragments are fragile, gentle pipetting is absolutely required at this step.

**Fig. 1:**
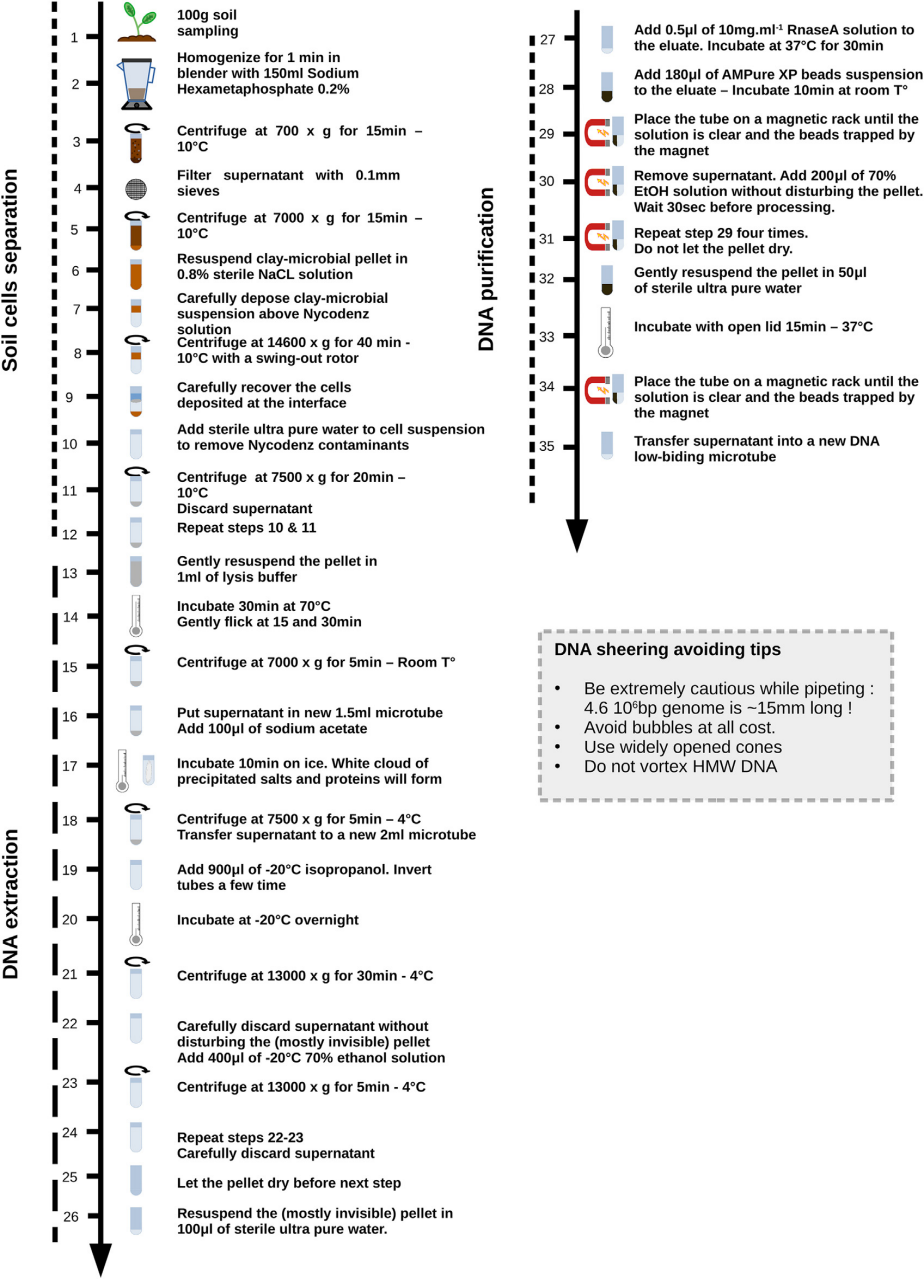
Nycodenz based graphical protocol. HMW = high molecular weight. Bp = base pair.

The complete protocol is summarized in Fig. 1.

This protocol is a modification of original DNA extraction protocol by Tournier et al. *[**22**]*. Modifications mainly lie in washing steps, centrifuge temperature and increase in number of wash steps from one to four. This allows for a greatly enhanced purification of DNA extracts, making them suitable for ONT full length 16S based metabarcoding and metagenomics sequencing.

## Protocol 2 – MP based DNA extraction

### Associated kit : MP fastdna© spin kit for soil

#### Protocol

**Sample preparation**

1. Weight 500 mg of soil to a Lysing Matrix E tube
2. Add 978 µL Sodium Phosphate Buffer to sample in Lysing Matrix tube
3. Add 122 µL MT Buffer

**DNA extraction**

4. Homogenize in the FastPrep© instrument (2 × 40 s at 6 m.s^−1^). Put in ice while instrument is resting.
5. Centrifugate at 14 000 g for 10 min – at 4 °C.
6. Transfer supernatant to a clean 1.5 mL microtube.
7. Add 250 µL of PPS solution, invert ten times. Put on ice for 10 min. *Ice incubation step helps protein and salts precipitation*
8. Centrifuge at 14 000 g for 5 min – at 4 °C.
9. Transfer supernatant in a 2 mL microtube
10. Resuspend the SiO_2_ DNA binding matrix and add 1 mL to the sample.
11. Place the tube on a rotator for 10 min (23 rpm) at room temperature.

**DNA purification**

12. Centrifuge at 14 000 x g for 2 min – room temperature. Discard supernatant. Room temperature allows for a better humic acid and other contaminants elution into buffer.
13. Resuspend the DNA binding matrix in 500 µl guanidine thiocyanate 5.5 M
14. Place the suspension in a Spin Filter column.
15. Centrifuge at 14 000 x g for 1 min – room temperature. Discard the guanidine thiocyanate solution.
16. Repeat step 13.
17. Centrifuge at 14 000 x g for 1 min – room temperature. Discard the guanidine thiocyanate solution.
18. Resuspend the binding matrix in 500 µl SEWS-M washing solution. We observed that the molecular grade 70 % EtOH solution can be used instead of SEWS-M solution, offering similar results.
19. Centrifuge at 14 000 x g for 1 min – room temperature. Discard the washing solution.
20. Repeat steps 18 to 19 four times. It is important to extract a DNA sample as pure as possible in the case you want to use this sample for metagenomics sequencing as well. Increase washing step numbers depending on humic acid residues.
21. Centrifuge the dry column at 14 000 x g for 2 min – room temperature. Discard the last drops of washing solution.
22. Resuspend the DNA matrix in 100 µl of sterile ultra pure water
23. Incubate the sample at 55 °C for 10 min to enhance DNA recovery
24. Transfer the column in a new DNA low binding microtube and centrifuge at 14 000 x g for 1 min – room temperature.
25. Add 180 µl of AMPure XP beads suspension to the eluate (= 1.8 ratio) – Incubate 10 min at room temperature. *Warming the bead suspension to 30* °*C beforehand can improve its efficiency. Resuspend the beads completely before pipetting. Humic acids being in competition with DNA for binding to the magnetic beads, the eluate must be as clear as possible. Note that sparQ PureMag Beads (QuantaBio) works with similar efficiency (ratio 1.8)*
26. Place the tube on a magnetic rack until the supernatant clears due to the migration of beads on the wall of the tube. *Magnetic rack, can be purchased or homemade 3D printed after downloading the 3D model designed for this application (*https://www.tinkercad.com/things/5kDi01FuJtp*). This model fits with Ø10* *mm and 2* *mm thick powerful magnets (such as N48, N52, neodymium magnets).*
27. Leave the tube on the rack, open the cap, remove and save supernatant (in case of error). Add 200 µl of molecular biology grade 70 % ethanol solution without disturbing the beads. Wait 30 sec before discarding supernatant.
28. Repeat four times step 27, then let ethanol residue dry with open cap but not to the point of cracking.

**DNA elution**

29. Gently resuspend the beads in 50 µl of sterile ultra pure water. High quality DNA can form a string of pearls when it resuspends. At the opposite, immediate resuspension of the beads may indicate a low amount or a highly fragmented state of DNA.
30. Incubate tubes with open caps for 15 min at 37 °C to eliminate ethanol residues. E*thanol might impair future sequencing reactions. Thermal block lid should be closed to avoid contamination.*
31. Place the tube back on the magnetic rack until the solution clears.
32. Carefully transfer the supernatant into a new DNA low-biding microtube. HMW DNA fragments are fragile, gentle pipetting is absolutely required at this step.

## Methods for protocol validation

### DNA integrity and purity

**Fig. 2:**
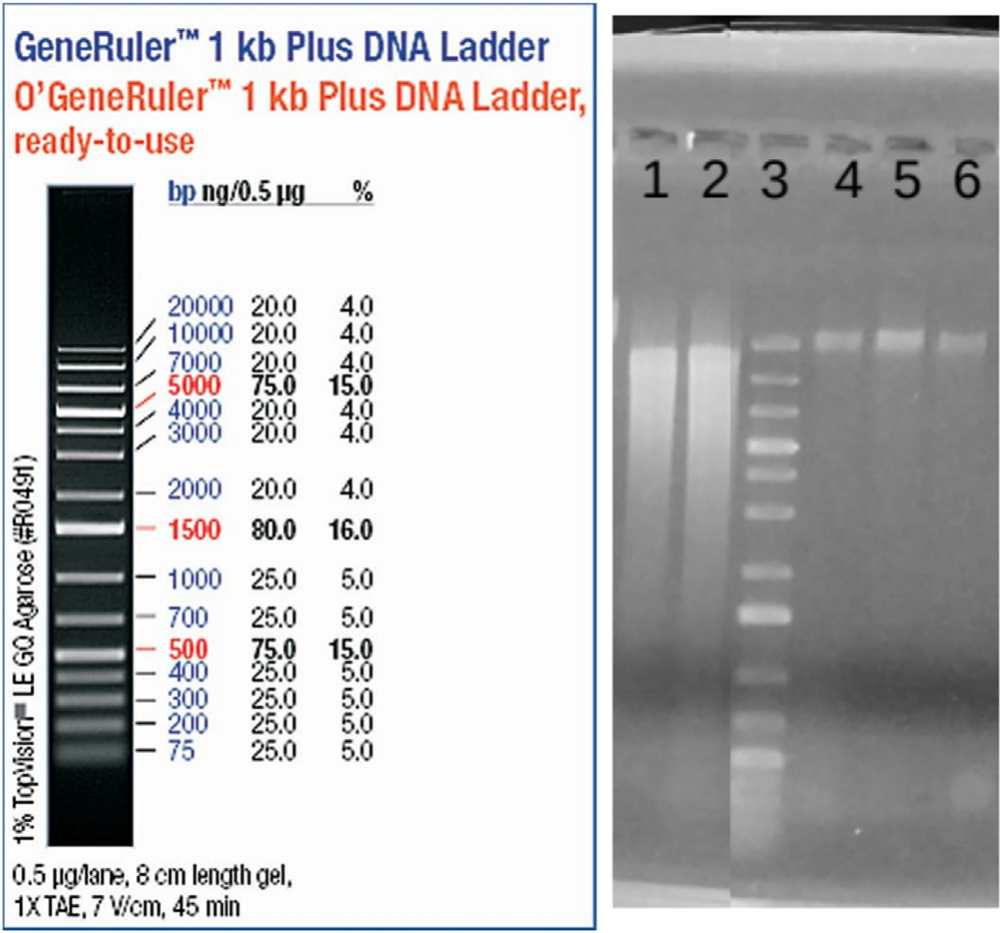
1 % Agarose gel with DNA samples from different protocols and treatments. 1 – Ultra Pure MP kit based DNA extract 2- Tournier et al. MP kit based DNA ; 3 - Ladder GeneRuler 1 kb+ ; 4 – Nycodenz based DNA extract before RNase treatment; 5 - Nycodenz based DNA extract after RNase treatment (61 µg.µl^-1^ 37 °C – 30 min) ; 6 – Final product : Nycodenz based Ultra Pure HMW DNA.

DNA integrity has been verified by electrophoresis. A volume of 5 µl of extracted DNA samples has been loaded on a 1 % Agarose gel, along with the GeneRuler 1 kb Plus DNA Ladder, 120 V, run during 40 min (Fig.2).

DNA recovery yield and purity have been quantified by the analysis of absorbance using an Eppendorf BioPhotometer and a Hellma TrayCell (Cat. No 105.800-UVS) adapter. The 260 nm absorbance was used to measure DNA quantity, while a ratio of 260/280 nm absorbance indicated either the presence of RNA when higher than ∼1.8 or the presence of proteins when lower than ∼1.8. A 260/230 nm absorbance ratio highly lower than 2.0–2.2 indicates the presence of contaminants such as salts and/or lysis buffer. DNA concentration was also assessed with fluorescence staining, using the Quant-iT™ PicoGreen™ dsDNA Assay Kits (Invitrogen) following the instruction of the manufacturer. Ratio of DNA concentration measured by PicoGreen on concentration measured by Nanodrop (Pico/Nano ratio) allows to quantify for proportion of 260 nm absorbance resulting from DNA concentration (or resulting from the presence of contaminants with absorbance at 260 nm, such as humic acids[23]). The closest to 1, the more 260 nm absorbance is due to the DNA presence regarding to contaminants with overlapping absorbances. RNase treatment is capital as RNA might impair subsequent Nanopore library preparation. AMPureXP beads purification treatment too as co-extracted pollutants (humic acids, salt, EDTA, protein etc.) can have a significant impact on sequencing run (“Input DNA/RNA QC” - IDI_S1006_v1_revB_18Apr2016 – Oxford Nanopore resources).

### Sequencing and N50 estimation

DNA samples were sequenced with Nanopore MK1C device and R9.4.1 technology flowcells using the Ligation Sequencing Kit (LSK-110) following Oxford Nanopore associated protocol (GDE_9108_v110_revL_10Nov2020, ONT internal ressources). Briefly, 1 µg of ultra pure DNA samples were end-repaired using NEBNext FFPE DNA Repair Mix (M6630) and NEBNext Ultra II End repair / dA-tailing Module (E7546) from the NEBNext® Companion Module for Oxford Nanopore Technologies® Ligation Sequencing kit with a 20 °C – 5 min followed by 65 °C – 5 min incubation on a thermocycler. Repaired DNA fragments were then purified using AMPure XP beads and washed with molecular biology grade 70 % ethanol prepared with MilliQ H2O. Pure repaired products were then ligated to sequencing adapters (AMX-F) with the T4 ligase from the NEB companion associated third-party reagents kit. In the second step of AMPure XP beads DNA purification, 2 × 250 µl of long fragment buffer (LFB) were used to clean and enrich the eluate in long DNA fragments. Library was finalized according to instructions without modification. DNA samples were sequenced for roughly 24 h Raw trace was basecalled automatically using MinKNOW 22.08.9 and Guppy 6.2.11. N50 was obtained through the MinKNOW software. At the time, only R9.4.1 flow cells were available. The R10 upgrade is now available and is expected to improve sequences quality and sequencing yield.

## Results – Protocols validation

### DNA integrity and purity

Migration patterns showed much higher shredding with the MP kit based protocol (Fig. 2: lanes 1 and 2) than the Nycodenz based-protocol (lanes 4, 5 and 6). Highly fragmented DNA will increase the proportion of short acquired DNA sequences. This has consequences regarding MAG reconstruction, repeated regions resolving, synteny confidence, especially for unknown genomes and organisms such as found in soil.

Both the Nycodenz and the MP based protocol allowed the recovery of ultra pure DNA products showing 260/280 ratios of ∼1.8, 160/230 ratios of ∼2 and Pico/Nano ratios of ∼1 (Table 2).

**Table 2:** DNA purity and sequencing size summary table. Tournier et al. (MP) represents the DNA extraction protocol published in [22], which lacks advanced purification methods and is not optimized for Nanopore sequencing. Ultra Pure MP corresponds to Protocol 2, which enhances the purity of DNA extracted using the Tournier et al. protocol while preserving its physical integrity. Ultra Pure Nycodenz represents Protocol 1, incorporating a soil cell separation step prior to DNA extraction to prevent physical fragmentation of cells and DNA. N50 (in kilobases) indicates the median length of the acquired DNA sequences. The 260/280 and 260/230 values are optical absorbance ratios used to assess DNA purity, with optimal values for UP DNA being ∼1.8 and ∼1.9–2, respectively.

Method	DNA Yield (µg of DNA per g of soil)	260/280	260/230	Pico/Nano	N50 (kbp)	External reference
Tournier et al. (MP)	20 - 30	1.8 – 1.9	0.01 – 0.1	0.4		Tournier et al
Ultra Pure MP	20 – 30	1.8	1.9 – 2	1	7.4 – 9.5	
Ultra Pure Nycodenz	0.05 – 0.1	1.8	1.8 – 1.9	1	14.27	

### DNA fragments median length (N50)

Acquired DNA sequences median length (N50) was used as a measure of the method ability to produce long DNA sequences. DNA sequences extracted by the MP kit showed N50 values around 9 kb. The purity is sufficient to perform ONT full length 16S metabarcoding. On the other hand, Nycodenz based DNA extracts showed a N50 of 14,27 kb and the longest sequence being 141,9 kb long. Despite low yields and known biases in sampling [24], Nycodenz based DNA extraction proved to be promising with extra pure HMW DNA extracts, which showed to be perfectly suitable for Nanopore shotgun metagenomics approaches, leading to the production of some ultra-long sequences and a N50 for bacterial soil DNA 3 to 7 times higher than what is usually found in the literature [[25], [26], [27], [28]]. Wick et al. 2010 [29] obtained slightly higher N50 (∼20 kb) but started from pure clonal culture which is not totally relatable to this work.

MinKNOWN sequencing software linked to Nanopore sequencing device displays useful data during a sequencing experiment, such as the reason that lead to the DNA fragment sequencing termination. We observed that long fragments (several kb) tended to stop being sequenced because of problems linked to their passage into the molecular pore (data not shown). This could be explained by DNA somehow spooling and blocking the pore as smaller fragments tended to pass all the way through the pore without problem. Consequently, the obtained N50 values may underestimate fragment lengths. N50 could improve if the entry of long fragments into the pore were facilitated, potentially benefiting from recent advancements in sequencing technology.

After this work was realized, Oxford Nanopore Technologies released a new sequencing library kit: the *Ultra-Long DNA Sequencing Kit V14 (SQK-ULK114)* that is supposed to be better suited for HMW DNA fragment correct pass through the pore. One of our perspective is to check in what proportion this kit can enhance N50 and read lengths in general. They also released a new sequencing chemistry: R10 flow cells, designed to produce more reads with higher accuracy. These were unavailable at the time, so sequencing our HMW DNA with this upgrade remains untested but could improve overall sequence quality and sequencing yield.

### Protocol comparison

Nycodenz based protocol is optimized to recover highly contiguous MAGs. It requires a large starting sample (e.g. 100 g of soil), which may be unsuitable for systems with limited soil amount available (e.g. rhizospheric soils). We do not recommend using the extracted DNA for metabarcoding assays as biased are known [24]. Nonetheless, this protocol is valuable for recovering long contiguous sequences from previously uncharacterized bacterial communities. Long contiguous sequences have proven to produce better results than short reads in almost every aspect linked to MAG recovery [11,12,16].

MP based protocol is more versatile, yields higher DNA amounts per gram of soil and requires smaller sample mass. It tends to recover shorter and less contiguous sequences than the other protocol This is suitable for both metabarcoding and metagenomics but reduces the potential for MAG assembly and synteny recovery if used alone.

These protocols can be effectively combined for metagenomic studies. Nycodenz based protocol can be used to recover global synteny through assembly, while the simplicity and scalability of the MP based protocol enable high-throughput sampling across multiple points. Mapping sequences from the second protocol to assemblies generated using first protocol provides differential coverage data, which is essential for accurate binning – a critical step in MAGs recovery.

### Troubleshooting

It exists a wide variety of soils with different mineral and chemical composition. Different soils than the one used for this protocol might show different results and behavior. If a soil is not meeting the purity standards for ONT sequencing, we advise increasing the number of ethanol washing steps. Increasing the number of ethanol washing steps while DNA is bonded to magnetic beads for the Nycodenz and MP based protocols, and increase the number of washing steps while DNA is bonded to the silica binding matrix for the MP based protocol.

## Conclusion

These two DNA extraction protocols focus on achieving high-purity DNA products suitable for ONT shotgun sequencing. The Nycodenz-based protocol allows for the recovery of ultra pure, HMW DNA, making it ideal for Oxford Nanopore shotgun metagenomics. The MP-based protocol yields ultra-pure DNA extracts with higher yields than the Nycodenz protocol, but at the cost of smaller fragment lengths.

## CRediT author statement

**Arthur Cousson:** Conceptualization, Methodology, Software, Validation, Formal Analysis, Investigation, Data Curation, Writing - Original Draft, Writing - Review & Editing, Visualization. **Anne-Laure Pablo:** Conceptualization, Methodology, Ressources. **Laurent Cournac:** Project Administration, Funding Acquisition. **Gabin Piton:** Ressource, Review & Editing. **Elisa Taschen:** Ressource, Review & Editing. **Damien Dezette:** Ressources. **Agnès Robin:** Ressources, Review & Editing. **Laetitia Bernard:** Conceptualization, Writing - Review & Editing, Visualization, Supervision, Project Administration, Funding Acquisition.

## Supplementary material *and/or* additional information [OPTIONAL]

You may also submit supplementary material with your article. This is not compulsory. If you do submit supplementary files, you are welcome to provide supporting details in this OPTIONAL section. More information is available in the Guide for Authors.

• ***Supplementary material*** *relates directly to the work that you have submitted and can include extensive Excel tables, raw data etc. We also encourage you to include failed protocols or describe adjustments to your protocol that did not work.*
• ***Additional information****can include anything that is not directly related to your protocol,* e.g. *more general background information, useful links etc. As the protocol article format doesn't feature an introduction, you might want to use this section to highlight information you would typically include in your introduction.*

## Declaration of competing interest

The authors declare that they have no known competing financial interests or personal relationships that could have appeared to influence the work reported in this paper.

## Data Availability

Data will be made available on request.
